# Creation of RTOG compliant patient CT-atlases for automated atlas based contouring of local regional breast and high-risk prostate cancers

**DOI:** 10.1186/1748-717X-8-188

**Published:** 2013-07-25

**Authors:** Vikram M Velker, George B Rodrigues, Robert Dinniwell, Jeremiah Hwee, Alexander V Louie

**Affiliations:** 1 Schulich School of Medicine and Dentistry, Western University, London, ON, Canada; 2 Department of Radiation Oncology, London Regional Cancer Program, London, ON, Canada; 3 Department of Epidemiology and Biostatistics, Western University, London, ON, Canada; 4 Department of Radiation Oncology, Princess Margaret Hospital, University Health Network, University of Toronto, Toronto, ON, Canada

**Keywords:** Radiotherapy, Breast cancer, Prostate cancer, Contouring, Atlas segmentation, Target volume delineation

## Abstract

**Background:**

Increasing use of IMRT to treat breast and prostate cancers at high risk of regional nodal spread relies on accurate contouring of targets and organs at risk, which is subject to significant inter- and intra-observer variability. This study sought to evaluate the performance of an atlas based deformable registration algorithm to create multi-patient CT based atlases for automated contouring.

**Methods:**

Breast and prostate multi-patient CT atlases (n = 50 and 14 respectively) were constructed to be consistent with RTOG consensus contouring guidelines. A commercially available software algorithm was evaluated by comparison of atlas-predicted contours against manual contours using Dice Similarity coefficients.

**Results:**

High levels of agreement were demonstrated for prediction of OAR contours of lungs, heart, femurs, and minor editing required for the CTV breast/chest wall. CTVs generated for axillary nodes, supraclavicular nodes, prostate, and pelvic nodes demonstrated modest agreement. Small and highly variable structures, such as internal mammary nodes, lumpectomy cavity, rectum, penile bulb, and seminal vesicles had poor agreement.

**Conclusions:**

A method to construct and validate performance of CT-based multi-patient atlases for automated atlas based auto-contouring has been demonstrated, and can be adopted for clinical use in planning of local regional breast and high-risk prostate radiotherapy.

## Background

Radiotherapy has become a standard of care in the radical treatment of high-risk prostate disease, and in the adjuvant treatment of locally advanced breast cancers. The delivery of radiotherapy has been improved through technological advances including intensity modulated radiotherapy (IMRT) and image-guided radiotherapy (IGRT), both of which have facilitated improved ability to deliver a given dose to target structures, while minimizing dose to organs at risk. Increasingly, IMRT has been utilized in the delivery of radiotherapy for cancers of the breast and prostate for normal tissue sparing and improved cosmetic outcomes[1]. However, utilization of these advanced treatment technologies requires a comprehensive and accurate understanding of the cross-sectional CT anatomy to accurately delineate the target and normal tissue structures.

The process of target and normal tissue delineation, known as segmentation or contouring, is subject to significant levels of inter- and intra-observer variability in both the accuracy and reproducibility of structures [2-8]. Accurate delineation of target volumes is critical to ensure adequate coverage and reduce the risk of local recurrence[4]. Currently, it is argued that inter-observer variability amongst radiation oncologists is the most significant contributor to the uncertainty in radiation treatment planning[9]. Contouring inconsistencies can negate the advantages of IMRT and may confound clinical trial results. Consensus contouring guidelines have been developed for high risk breast and prostate cancers to guide the delineation of the primary and lymph node clinical target volumes, and reduce overall inter-observer variability [4,10-12]. Manual target volume delineation is also more time consuming than traditional planning. Time efficiency and contouring variability present two potential barriers to the uptake and utilization of IMRT for breast and prostate cancer radiotherapy.

A recent technological development in radiation treatment planning is the use of automated atlas-based segmentation (AABS) algorithms to aid in the delineation of target volumes'. Atlas' refers to a model set of expertly contoured CT images for one (single-patient) or more (multi-patient) cases that serve to guide delineation of target volumes for similar cases. Computerized AABS algorithms utilize CT-based atlases as a template to deformably register and auto-segment new cases. A proposed advantage of multi-patient atlas is the software can scan a database of pre-contoured patients to find one with similar anatomical variation to the new patient to be contoured, potentially improving the accuracy of auto-segmentation. Studies evaluating AABS algorithms in multiple disease sites have previously demonstrated the potential to improve efficiency and variability associated with manual segmentation [13-27].

The purpose of this study was to construct a multi-patient CT-based atlas of expertly contoured high-risk regional nodal breast and prostate cancer cases, and to evaluate the performance of a commercially available AABS for efficiency and accuracy in delineation of clinical target volumes and organs at risk.

## Methods

An anonymized CT-database of 124 left and right sided post-lumpectomy breast cancer patients and 25 high risk prostate cancer patients simulated for treatment at the London Regional Cancer Centre between January and December of 2009. This database, part of the LocStar (London Ontario Consistently Segmented Tri-purpose Atlas Resource) project, was utilized to randomly select 25 left breast, 25 right breast, and 14 prostate cancer cases. Ethics approval for this patient database was obtained from the University of Western Ontario REB. CT image datasets were stored according to the Digital Imaging and Communications in Medicine (DICOM) standards of practice. Breast cancer patients were simulated supine, arm above head immobilized on a breast board, while prostate patients were simulated supine with full bladder using standard technique. Patients with pacemakers, breast implants, or hip prostheses were excluded. CT scans were non-contrast enhanced with 3 mm slice thickness.

Post-lumpectomy high risk breast cancer cases were contoured by a trained Radiation Oncology resident (VV) with contours reviewed by an expert breast Radiation Oncologist (RD) to ensure conformity with the RTOG Consensus Guidelines for breast radiotherapy[28]. The CTVs for whole breast and chest wall (CTV_BRCWL_), supraclavicular lymph nodes (CTV_SCV_), internal mammary nodes (CTV_IMN_) and axillary levels I, II, and III (CTV_AXI,_ CTV_AX2,_ CTV_AX3_) were contoured according to RTOG guidelines[28]. Additionally, the lumpectomy cavity (LUMP), and the organs at risk, heart (H), left lung (LL), and right lung (RL) were delineated. Using the British Columbia Seroma Clarity Scale, a score of 1 to 5 was assigned to denote the confidence of the lumpectomy cavity delineation [29].

High-risk prostate cancer cases were contoured by an expert genitourinary Radiation Oncologist (GR) according to the RTOG guidelines. CTV for the prostate (CTV_PROS_), pelvic nodes (CTV_NODES_) and OARs of rectum (R), bladder (B), penile bulb (PB), left femur (LF) and right femur (RF) were delineated.

Contouring and subsequent atlas-construction were performed utilizing a commercially available, multi-patient atlas AABS capable software suite (MIM Version 5.2, MIMVista Corp, Cleveland, Ohio). Manual contouring was performed using contouring tools including slice-by-slice deformable registration. The time to manually delineate each structure was recorded. This created a data set of 25 left breast, 25 right breast, and 14 prostate gold standard contoured cases.

**Figure 1:**
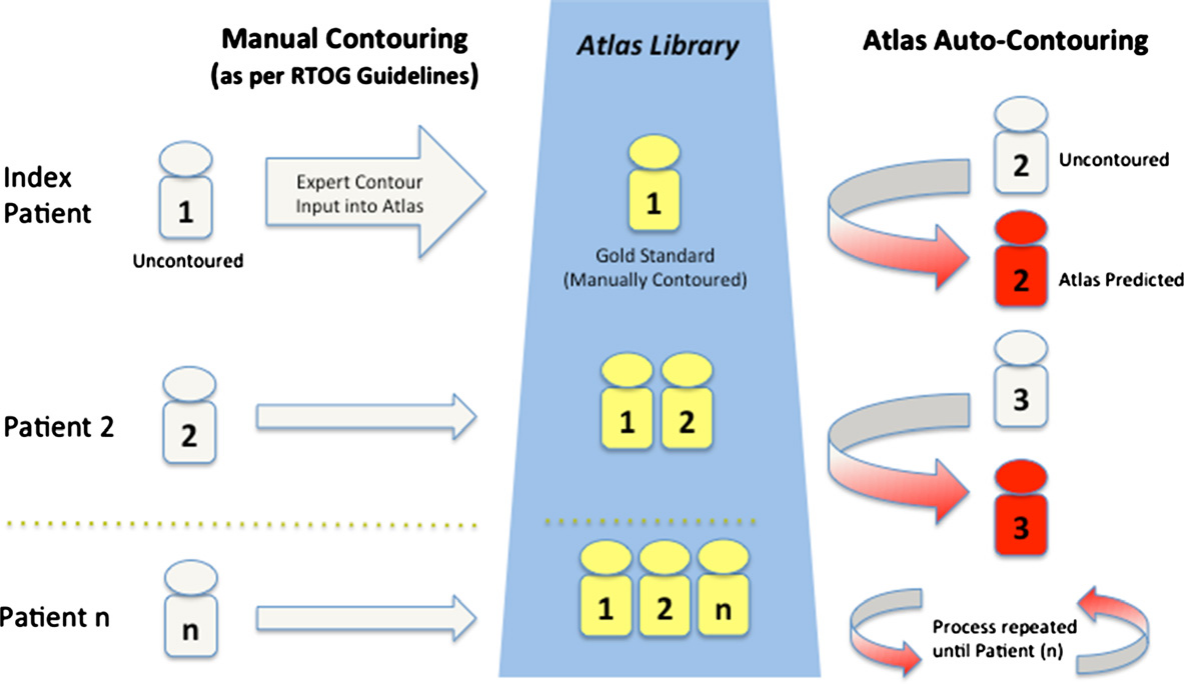
Atlas building process map.

The atlas building methodology is illustrated in Figure 1. The left-breast post-lumpectomy atlas was constructed first. The atlas builder (VV) randomly selected and manually contoured the index case. This Patient #1 (P1) was added to the atlas library, such that the library now contained one case (n = 1). Patient #2 (P2) was then chosen at random to have the AABS algorithm create a set of automated atlas-predicted contours. Since the atlas only contained P1, the algorithm selected P1 as the best match. The CT images of P1 were registered to fit the uncontoured CT images of P2. The contoured structures on P1 (CTVs and OARs) were then deformably registered to fit P2’s anatomy, creating a set of automated atlas predicted contours, which were stored as P2_PREDICTED_.

Next, the manually contoured (gold standard) P2_GOLD_ was added to the atlas library, now containing two cases (n + 1), P1_GOLD_ and P2_GOLD_. The AABS was again used to create automated contours for Patient #3 (P3), now able to choose between the two atlas patients (P1_GOLD_ and P2_GOLD_) to find the best match for anatomy for P3. The atlas predicted contours P3_PREDICTED_ were saved, and then the manually contoured P3_GOLD_ was added to the atlas. In this fashion, atlas building continued for all 25 patients, which yielded 24 patients with manually delineated contour P(n)_GOLD_ and a atlas predicted P(n)_PREDICTED_ contour set for statistical comparison. This atlas building and validation method was repeated for the right breast post-lumpectomy and high risk prostate cases.

### Statistical analysis

Statistical comparison of manually delineated versus atlas predicted contour sets was performed using StructSure (Standard Imaging, Wisconsin, USA) to calculate the dice similarity coefficient (DSC). The DSC metric is a simple spatial overlap index for volumetric comparison, defined as:

$$\left(V_{1},V_{2}\right)=|2|V_{1}\cap V_{2}|\;/\;|V_{1}|+V_{2}$$

where V_1_ and V_2_ are the respective volumes of the first and second contours, and ∩ is the intersection. It follows that a DSC of 1 equals perfect agreement (overlap), and a DSC of 0 equals no agreement. DSC for each region of interest (ROI), contouring times, and seroma scores were reported using descriptive statistics. Logit transformation of the DSC, such that the score followed a normal distribution, was performed for quartile ANOVA analysis and student t-test to determine if atlas performance improved with increased case number.

## Results

### High risk breast cancer post-lumpectomy

The high risk left and right-sided breast cases were analyzed separately. The mean AABS segmentation time (SD, range) for a single case was 86 s (9, 69-102 s) and 87 s (13, 60-113 s) for left and right, respectively. No statistically significant difference in automated segmentation time was found with increased atlas patient number. Mean manual segmentation time (SD, range) for a single case was 3177 s (387, 2559-4166 s) and 2905 s (2450 – 3433 s) for left and right, respectively.

**Figure 2:**
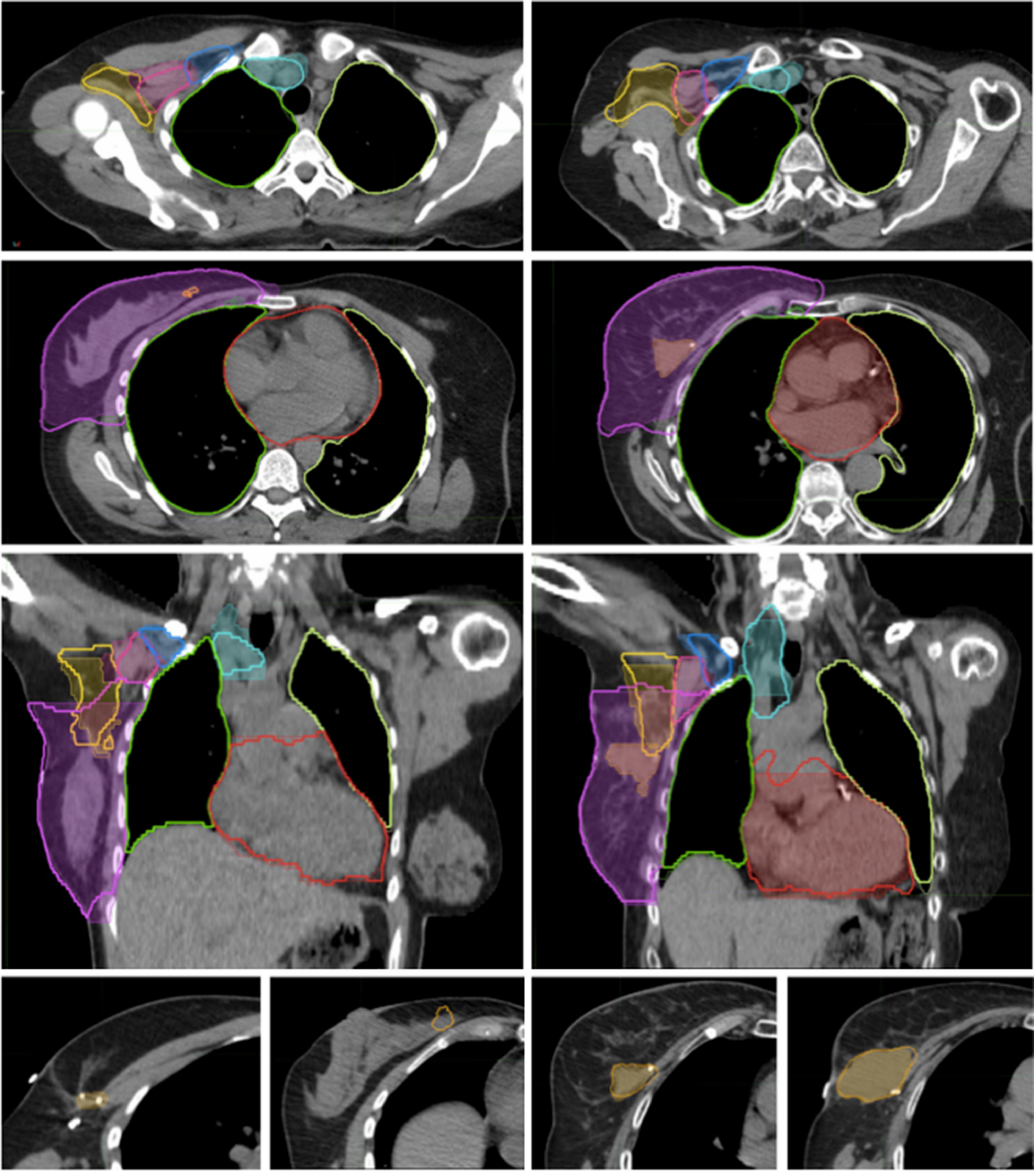
**Atlas-predicted contouring for high risk breast cancer post lumpectomy.** Representative slices of two contoured right breast-post lumpectomy cases comparing atlas predicted auto contours (bold outline) to manual contoured standard (thin outline with colourwash). Atlas subjects #5 (left images) and #14 (right images). ROIs demonstrated are CTV_BRCWL_ (purple), CTV_AX1_ (yellow), CTV_AX2_ (pink), CTV_AX3_ (dark blue), CTV_SCV_ (teal), LUMP (orange), H (red), LL(light yellow), RL(green). Bottom panels demonstrate the high degree of variability in seroma (LUMP) prediction with the atlas being unable to predict the subtle seroma (left images, DSC = 0) while contouring a more readily identifiable seroma with reasonable accuracy (right images, DSC = 0.89).

Means DSC scores and ranges of targets are reported in Table 1. Good levels of agreement (mean DSC 0.80 to 1) were demonstrated (mean DSC left and right post-lumpectomy respectively) for CTV_BRCWL_ 0.87 and 0.89 and organs at risk of LL 0.97 and 0.97, RL 0.97 and 0.97, and H 0.89 and 0.90. Moderate levels of agreement (mean DSC 0.60-0.79) were demonstrated for nodal targets CTV_AXI_ 0.72 and 0.75, CTV_AX2_ 0.74 and 0.73, CTV_AX3_ 0.69 and 0.73, CTV_SCV_ 0.72 and 0.70. Poor levels of agreement (DSC < 0.60) were demonstrated for CTV_IMN_ 0.25 and 0.33 and LUMP 0.10 and 0.04. Two representative breast cases are depicted in Figure 2.

**Table 1:** DSC comparison of atlas predicted ROIs to manually contoured gold standard for the locoregional breast cancer atlases

**Contoured structure**	**ROI name**	**Left breast post lumpectomy (mean DSC, SD, range)**		**Right breast post-lumpectomy (mean DSC, SD, range)**	
Breast and Chest wall	CTV_BRCWL_	0.87	(0.03, 0.80–0.92)	0.89	(0.04, 0.79–0.94)
Axilla I	CTV_AX1_	0.72	(0.09, 0.43–0.86)	0.75	(0.08, 0.59–0.87)
Axilla II	CTV_AX2_	0.74	(0.06, 0.62–0.87)	0.73	(0.06, 0.63–0.84)
Axilla III	CTV_AX3_	0.69	(0.08, 0.52–0.80)	0.73	(0.07, 0.54–0.83)
Supraclavicular	CTV_SCV_	0.72	(0.07, 0.57–0.82)	0.70	(0.08, 0.52–0.82)
Intramammary Nodes	CTV_IMN_	0.25	(0.14, 0.01–0.59)	0.33	(0.15, 0.03–0.58)
Lumpectomy Cavity	LUMP	0.10	(0.20, 0–0.69)	0.04	(0.11, 0–0.89)
Heart	H	0.89	(0.03, 0.81–0.92)	0.90	(0.04, 0.81–0.95)
Left Lung	LL	0.97	(0.01, 0.96–0.98)	0.97	(0.01, 0.95–0.98)
Right Lung	RL	0.97	(0.01, 0.95–0.99)	0.97	(0.01, 0.96–0.98)

N = 25 Left-, 25 Right- High Risk Post Lumpectomy Cases.

ANOVA quartile analysis showed a statistically significant improvement in DSC scores for left breast post-lumpectomy LL (Quartile 1 to 4, 0.967 to 0.975, p = 0.03), RL (Quartile 2 to 4, 0.967 to 0.978, p = 0.03), and right breast post-lumpectomy CTV_SCV_ (Quartile 1 to 2, 0.608 to 0.724, p = 0.004). No statistically significant correlation between BC Seroma Score and LUMP DSC was found.

### High risk prostate cancer

For the high-risk prostate atlas, mean AABS segmentation time (SD, range) for a single case was 134 s (23, 106-179 s). No statistically significant difference in automated segmentation time was found with increased atlas patient number. Time for manual contouring of prostate cases (SD, range) was 354 s (81, 241-525 s).

**Figure 3:**
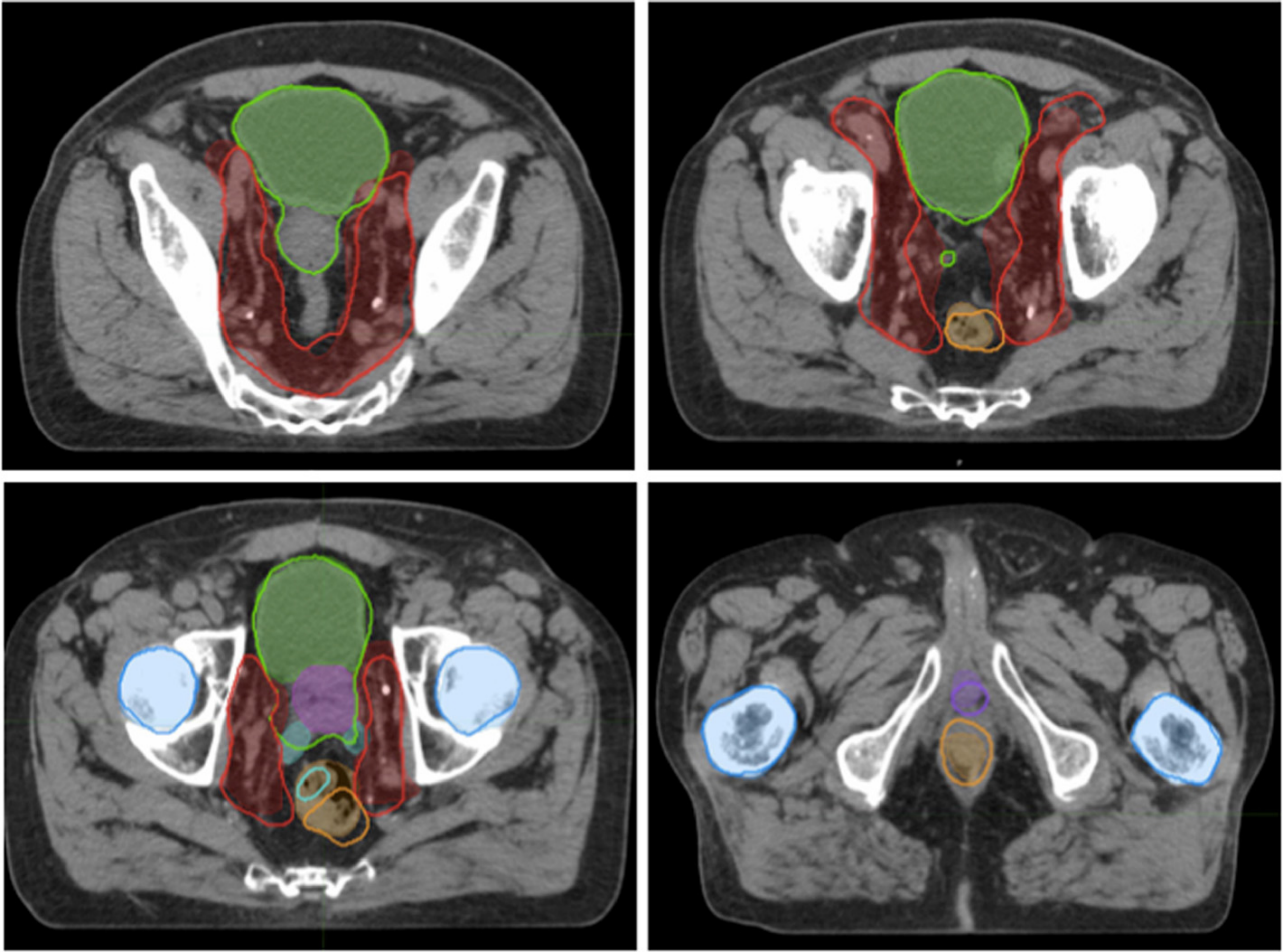
**Atlas-predicted contouring for high risk prostate cancer.** Sample slices of contoured high risk prostate cases comparing atlas predicted auto contours (bold outline) to manual contoured standard (thin outline with colourwash). ROIs demonstrated are CTV_PROS_ (purple), CTV_NODES_ (red), B (Green), R (Orange), PB (dark purple), SV (cyan), LF and RF (dark blue).

Means DSC scores and ranges of targets are reported in Table 2. Good levels of agreement (mean DSC 0.80 to 1) were demonstrated for B 0.84, LF (0.89) and RF (0.93). Moderate levels of agreement (mean DSC 0.60-0.79) were demonstrated for CTV_PROS_ 0.71 and CTV_NODES_ 0.71. Poor levels of agreement (mean DSC < 0.60) were demonstrated for R 0.48, PB 0.39, and SV 0.30. No statistically significant difference in t-test p-value between the first half and second half of cases added to the atlas (all p > 0.1). A representative prostate case is depicted in Figure 3.

**Table 2:** DSC comparison of atlas predicted ROIs to manually contoured gold standard for high risk prostate cancer atlas

**Contoured structure**	**ROI name**	**High risk prostate (mean DSC, SD, range)**	
Prostate	CTV_PROS_	0.71	(0.12, 0.35–0.84)
Pelvic Nodes	CTV_NODES_	0.71	(0.07, 0.58–0.79)
Bladder	B	0.83	(0.10, 0.63–0.95)
Rectum	R	0.48	(0.19, 0.17–0.76)
Seminal Vesicles	SV	0.30	(0.31, 0–0.79)
Penile Bulb	PB	0.39	(0.22, 0–0.67)
Left Femoral Head	LF	0.89	(0.09, 0.63–0.95)
Right Femoral Head	RF	0.93	(0.03, 0.86–0.96)

N = 14 High Risk Prostate Cancer Cases.

## Discussion

Adoption of AABS has the potential to improve the efficiency of workflow and segmentation conformity with consensus guidelines. This is the first study to test a multi-patient CT-atlas based auto-segmentation approach for local regional breast and high risk prostate cancers, two of the most common high volume disease sites treated by radiation oncologists. We have demonstrated a method to construct a multi-patient CT-based contouring atlas and have validated the performance of a commercially available auto-segmentation algorithm for these disease sites.

In the local regional breast scenario tested, the algorithm performed well for lung and heart, and these contours could likely be used with no, or minimal editing. For breast and chest wall, agreement was acceptable with high mean DSC scores (0.87 and 0.89), the majority of variation occurring in the posterior lateral border, where the medial edge of the latissimus dorsi provides a partial anatomical landmark. These results are consistent with a single-patient atlas based auto-segmentation study of whole breast by Reed et al. [19] which reported DSC of 0.94. Manual correction was required in the inferior and superior slices, yet auto-contouring of the breast surface and posterior chest wall were highly accurate and would likely not require any editing to be clinically appropriate.

Automated lymph node contours demonstrated modest levels of agreement, and require manual revision, especially for the designated supraclavicular compartment. Results for lymph node contouring were consistent with the modest levels of inter-observer agreement amongst experts in previous contouring studies [30], and can be interpreted as superior to the mean percentage overlap of the RTOG consensus guideline project by Li et al. [4]. There were low levels of conformity for highly variable and small volume structures such as the IMC chain and lumpectomy cavity. These delineations are based on judgment and subtle anatomical cues, and it is advised that these structures be contoured manually [25].

The use of a multi-patient approach to account for anatomical variation is different than the approach used by Anders et al. [25], where 3 separate individual atlases on the basis of breast volume (small, medium, large) were manually matched prior to AABS. They reported that breast shape, and not absolute volume, appeared to be more important and that a STAPLE (simultaneous truth and performance level estimation) contour generated from nine random patients was a superior template [25]. We have demonstrated minor improvements in lung contouring with increasing patient number, but no statistically significant improvement in prediction of target volume delineation was found beyond an atlas size of 12 individuals. This perhaps suggests an upper limit to the ability of the software algorithm to register the CT anatomy, and the number of cases required to benefit from the multi-patient atlas approach.

For high-risk prostate cases, the algorithm performed well for auto-delineation of the bladder and femurs. Predicted bladder contours were most variable at the bladder neck. For the rectum and penile bulb, conformity was low, with ambiguity of the superior and inferior limits of contouring. Prostate and pelvic nodal targets had moderate agreement, but would require manual correction and discretionary inclusion of a portion of the seminal vesicles. Qualitatively, prediction of pelvic nodal contours preserved the pattern of delineation in the consensus atlas and respected bony radial borders, however manual revision of iliac and pelvic vessels margins was required. A significant performance improvement with increased case number was not observed.

This study brings to issue the limitations of AABS and evaluation of contouring studies. We assigned a single expert to delineate the structures based on the RTOG consensus atlas, as the primary objective was to test pure algorithm performance, and intra-observer variation is generally less of a factor than inter-observer variability. In addition, although the current data set is from a single institution, standard simulation techniques were utilized and as such we believe will be applicable in other centers as well. As previous investigations have demonstrated that variances in segmentation have significant dosimetric consequences [4], it is recognized that only segmentation was assessed in this study. Although RTOG guidelines were utilized, this atlas building and evaluation method can be utilized by any clinician to compile and create an atlas of their cases to reflect their individual contouring practices.

Although we have utilized the DSC, a volumetric based concordance index, there is no consensus in the literature as to the appropriate metric for the analysis and comparison of contours [31]. Limitation of DSC is sensitivity to variability for small volume structures and that it does not quantitatively describe where within the segmented volume the majority of the variation occurs, assigning equal importance to all voxels. This is particularly evident in the comparison of large structures (ie. breast/chest wall) where the majority of volumetric deviation was in the superior and inferior slices, while intermediary slices were contoured with high fidelity. The integration of recently developed penalty metric scores that penalize for contouring variation near critical structures where the dosimetric consequence is more severe may improve on this metric in future studies [32].

We have demonstrated that the use of AABS algorithm for deformable registration is computationally time efficient. Although the time to correct auto-contours by multiple observers was not captured, and would be expected to vary widely, the MIM AABS tool has been previously demonstrated in head and neck and prostate bed contouring to afford significant time savings [17,22]. There is a question as to whether the use of AABS will bias a radiation oncologist’s contours, though our group has previously demonstrated, in the post-prostatectomy scenario, acceptable intra-observer agreement between edited auto-contours and manually generated contours, and lower levels of inter-observer variability [22]. Thus, AABS may hold promise for future application in clinical trials to reduce contouring variability amongst centres. A potential criticism of AABS is that while we believe an atlas based on the RTOG guidelines would be expected to improve accuracy by reducing the variability amongst multiple observers, we cannot confidently say that the guidelines do not bias away from the ‘truth’. Therefore, it is imperative that when auto-contouring is utilized, the contours must be reviewed and edited by a skilled radiation oncologist with an understanding of the patient’s clinical and pathological context.

## Conclusions

This is the first study to demonstrate the building of RTOG guidelines compliant CT-based multi-patient atlases for local regional breast and high risk prostate cancer treatment planning. Based on this performance evaluation of the MIM AABS algorithm, we recommend that it be considered for clinical use in the routine delineation of the lungs, heart, and bilateral femora with no editing required. Clinical target volumes generated for the breast/chest wall, axillary lymph nodes, prostate and pelvic nodes require review and editing by a radiation oncologist prior to clinical use. Bladder and rectum contours should be reviewed and may require minor editing. Small, highly variable structures such as lumpectomy cavities, the IMC chain, penile bulb, and seminal vesicles should be contoured manually. AABS algorithms hold considerable promise to improve efficiency of the contouring process and improve conformity with guidelines amongst multiple observers, although further advances in these technologies will continue to require rigorous performance evaluation prior to their adoption in clinical use.

## Abbreviations

RTOG: Radiation therapy oncology group; AABS: Automated atlas-based segmentation; IMRT: Intensity modulated radiotherapy; CTV: Clinical target volume; OAR: Organ at risk; DSC: Dice similarity coefficient; AX: Axillary compartment; SCV: Supraclavicular compartment; IMN: Internal mammary nodes; LL: Left lung; RL: Right lung; H: Heart; LUMP: Lumpectomy cavity; BRCWL: Breast/Chestwall; B: Bladder; R: Rectum; LF: Left femur; RF: Right femur; PB: Penile bulb; SV: Seminal vesicles.

## Competing interests

The authors declare that they have no competing interests.

## Authors’ contributions

VV carried out the breast contouring, statistical contour comparison, participated in interpretation of data, and drafted the manuscript. GR completed the prostate contouring, participated in interpretation of data and manuscript drafting, and helped conceive the study. RD reviewed the breast contours for accuracy and participated in manuscript editing. JH participated in the statistical analysis of data and editing of the manuscript. AL participated in study conception, interpretation of data, and manuscript editing. All authors read and approved the final manuscript.
